# Development of Open Backend Structures for Health Care Professionals to Improve Participation in App Developments: Pilot Usability Study of a Medical App

**DOI:** 10.2196/42224

**Published:** 2023-04-13

**Authors:** Florian Dittrich, Urs-Vito Albrecht, Julian Scherer, Sören L Becker, Stefan Landgraeber, David Alexander Back, Kai Fessmann, Marcel Haversath, Sascha Beck, Mona Abbara-Czardybon, Henning Quitmann, Anna Katharina Harren, Matthias Aitzetmüller, Marie Luise Klietz

**Affiliations:** 1 Department for Orthopaedics and Orthopaedic Surgery Saarland University Homburg Germany; 2 Department of Digital Medicine Medical Faculty Ost-Westfalen-Lippe Bielefeld University Bielefeld Germany; 3 Joint Centre Bergisch Land Remscheid Germany; 4 Department of Trauma Surgery University Hospital of Zurich Zurich Switzerland; 5 Orthopaedic Research Unit University of Cape Town Cape Town South Africa; 6 Center for Infectious Diseases, Institute of Medical Microbiology and Hygiene Saarland University Homburg Germany; 7 Clinic of Traumatology and Orthopedics Bundeswehr Hospital Berlin Berlin Germany; 8 Clinic of Trauma, Hand and Reconstructive Surgery University Hospital Essen Essen Germany; 9 Clinic for Orthopaedics Hospital Nettetal Nettetal Germany; 10 Sportclinic Hellersen Hellersen Germany; 11 Division for Plastic Surgery, Department for Traumatology and Hand Surgery University Hospital Münster Münster Germany

**Keywords:** smartphone, mHealth, backend, usability, UX, user experience, mHealth, mobile health, health app, mobile app, app development, no-code

## Abstract

**Background:**

Efficient digitization in medicine still is in its infancy but undeniably has great potential for current and future challenges in health care. Thus far, the rollout of medical apps has not resulted in widespread use of smartphones in the German health care sector—the reasons for this have not been clarified so far. Nevertheless, the lack of user involvement in the development process and content creation might contribute to low acceptance of these products.

**Objective:**

This study aims to outline an approach to involve medical expertise without any coding knowledge for developing medical app content and functions.

**Methods:**

An end user–operable backend was built. Its usability was evaluated using a usability evaluation test protocol. The results of the usability tests were evaluated by the app development team, and the usability test was repeated for optimizing backend usability. In total, 40 criteria to measure the ease of app usage were defined a priori. The usability test comprised 20 tasks that had to be fulfilled. Usability tasks were analyzed for completion, dropout, and test duration. Due to the COVID-19 pandemic, digital videoconferencing platforms (Zoom and QuickTime Player) were used to complete usability questionnaires. Finally, several backend-based apps for several specialties (infectiology, plastic and reconstructive surgery, and orthopedics) were developed by health care professionals as prototypes.

**Results:**

Initial usability testing was conducted with 5 participants (4 men and 1 woman; mean age 39.2, SD 5.97 years). All of them could complete the assigned backend tasks with only a few workflow interruptions and some minor errors. After usability optimization, the workflow completion time decreased from 5.03 minutes to 3.50 minutes, indicating a time saving. The basic backend structure was clear to all test users and the handling was intuitive to learn. Some minor errors in the backend occurred during the test rounds. The apps developed using the aforementioned approach are in clinical use as a proof of concept.

**Conclusions:**

Backends offering operability for medical professionals might have great potential for app development in the mobile health sector. Sophisticated and time-saving usability are pivotal for the acceptance of medical software, as illustrated by the backend-based apps presented herein, which are in clinical use as a proof of concept. Basic interventions are essential and sufficient for adequate usability optimization. Practicable, well-structured software usability evaluation is possible based on the usability evaluation test protocol.

## Introduction

### Background

Since the introduction of smartphones in 2007, they have rapidly gained popularity and are omnipresent nowadays [1]. Daily use of smartphone apps has become common for the purposes of communication, mobile payments, and booking appointments [2]. In a technical sense, apps are small programs that offer specific functions by providing an (preferably) intuitive user interface, which is often referred to as the “frontend” [3]. A “backend” directly contrasts the frontend, representing the corresponding part of an app that stores, secures, and processes data or codes that interpret program syntaxes. App backends are managed by the administrator and are inaccessible to end users [4]. An example of backend use is its usage as a data content management system (DCMS) in processing and digitizing larger data sets, allowing user-friendly access to end users on their smartphones via a connected app (frontend) [5].

Digitization in medicine is still lacking but undeniably has major potential for current and future challenges in health care [6,7]. Political, legal, and structural frameworks for implementing digital solutions present various challenges for health care systems [8]. After initial ground-breaking steps, the German legislature finally gathered pace toward a stringent national digitization strategy [9]. The Digital Healthcare Act paved the way for prescriptions of digital health apps, use of web-based video consultations, and improved data security in health data communication [10]. The Digital Healthcare Act has offered a new perspective on high social demands requiring digitization and smartphone implementation in medical treatment [11]. This entitlement has initially been limited for statutorily insured Germans to low-risk (class I and IIa) medical devices, which have been included by the German Federal Institute for Drugs and Medical Devices in the publicly accessible register for digital health apps [12,13]. However, these regulatory measures have not led to the intended widespread smartphone implementation in everyday treatment so far [14]. Regardless of underlying causes, it can be assumed that the lack of user involvement in the development process might contribute to low acceptance of medical apps among health care professionals [15]. In contrast to this, the society that adapts increasingly to digitization expects integration of apps in the health care sector [16].

Apps focusing on the flow of information to optimize patient education might help to support patients in their health issues. However, only few medical professionals (MPs) and patients reported previous medical app usage [17]. Satisfactory and affordable app merchandizing seems lacking [18]. As shown before, medical app development using a backend-based, app-corresponding DCMS, has proven to be highly effective and adaptable to specific users’ requirements or varying standards [19].

### Objective

To increase the involvement of health care professionals in app development, this study outlines an approach to develop app content and function without any coding knowledge. As a first step, a specific methodology was established to ensure end user operability of the backend by developing a user-friendly interface. As a second step, multiple app prototypes in use by health care professionals and patients were developed with the backend-based DCMS—these are presented as a proof of concept. The introduction of the potential and functionality of backends might inspire more adaptable mobile health (mHealth) solutions for digitizing the health care sector.

## Methods

### Development Protocol

#### Basic Software Conception

A multidisciplinary team was involved in the development process of the software. The team comprised 2 physicians (FD and SB), a lawyer, a user experience (UX) designer, and a software and web developer. The backend server runs on a web-based app based on the hypertext preprocessor framework Symfony and meets software security guidelines (eg, the International Organization for Standardization/ International Electrotechnical Commission standard 27001 of 2013, standard 27017 of 2015, standard 27018 of 2019, standard 27701 of 2019, and standard 9001 of 2015). All server structures are located in Germany. No patient-related data were obtained. The backend works as a DCMS and supports the digitization of larger data sets. Different functions can be performed via the backend that directly modifies the linked app’s design and functionality (frontend). For example, not only text but also font size, style, and color can be added or changed in the backend. Links, images, and videos can also be integrated. Any data exchange between the backend and apps runs via a secure sockets layer–secured connection. DCMS-corresponding apps were developed using React Native (Facebook Inc) technology. React Native is a Javascript-based framework for software developers, building cross-platform mobile apps for Android or iOS devices. The framework features built-in components and application programming interfaces, which are essential for developing innovative and user-friendly mobile apps [20].

#### UX Optimization of the DCMS

**Figure 1:**
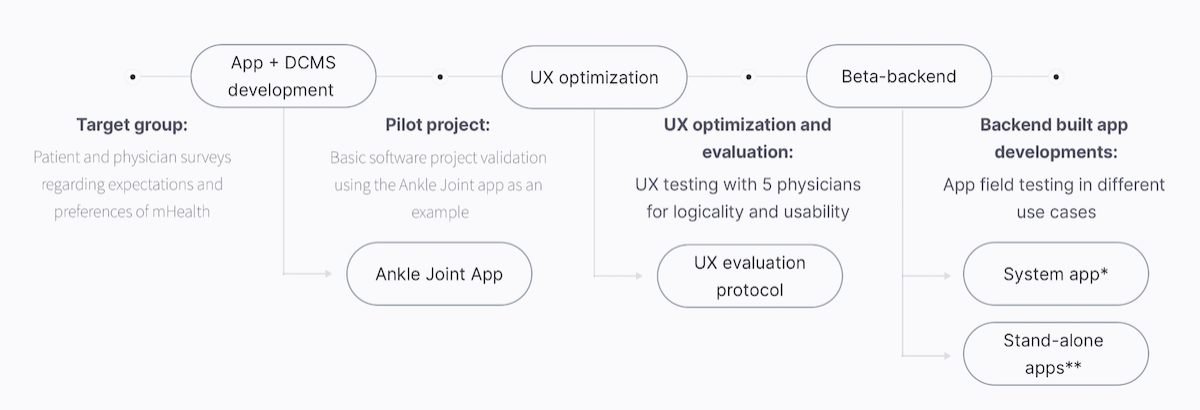
The DCMS development flowchart. *Backend-based stand-alone apps include individual functions, a unique app logo, and the implementation of individual corporate designs. The development process is more efficient than the alternative of “starting from scratch” with every app because a modular build system can be used. **A contentless system app with predefined functions, which communicates with the backend, is even simpler. The empty app could be adapted to the respective requirements via the backend and transferred to the system app via QR codes or links, which would then personalize itself. DCMS: data content management system; mHealth: mobile health; UX: user experience.

**Figure 2:**
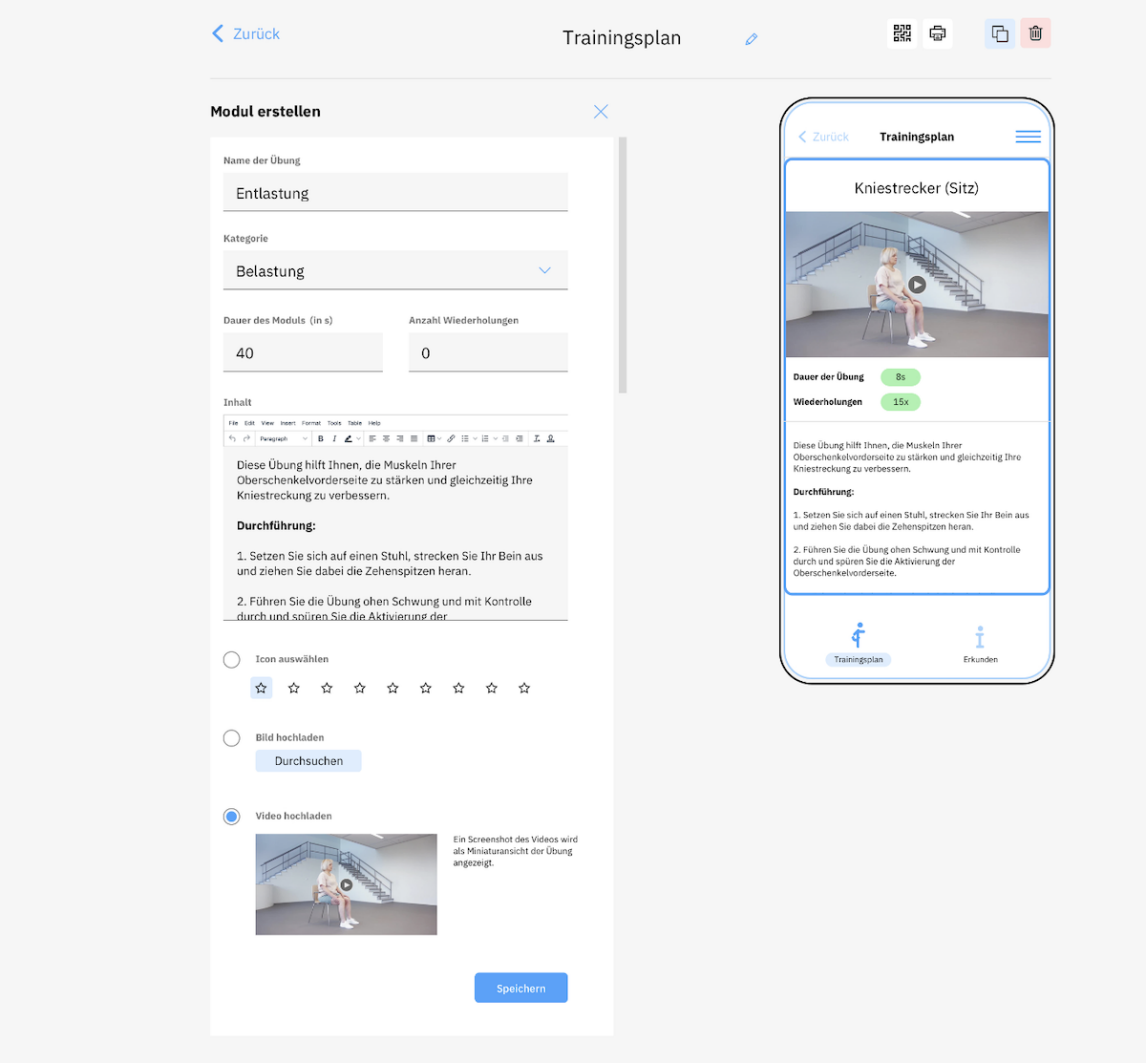
Visualization of one action path for the test users by using templates. The mechanism from the content edit box to the in-screen simulation is shown.

Before starting the software development process, patients’ and physicians’ preferences for medical apps were elicited [17,18]. In consideration of the survey results, a digital follow-up treatment plan for ankle joint sprains was created. To evaluate the basic software concept, the Ankle Joint App was developed using the DCMS system. It was successfully validated subsequently in a pilot project with the appropriate target group (Figure 1) [19]. In order to create an intuitive user interface and to enable MPs to work with the system, a user-friendly intermediate program mediating front- and backend activities had to be established. Based on predefined target points, which the physicians must fulfill independently via the backend to create app content, different specific wireframes were created. The action paths were visualized using templates and presented to a group of 5 physicians (Figure 2). A coherent design and a logical, consistent layout architecture were used for a clear presentation of the beta backend’s functions to the end users. The systematics of colors, logos, wording, and layout were evaluated for logic and coherence. After this first feedback round, change requests were discussed within the development team. Templates were changed appropriately, and programming of the backend beta version was initiated.

#### UX Evaluation Test Protocol

After completion of the beta backend programming, a UX evaluation test protocol was developed to validate the backend’s usability by MPs (Multimedia Appendix 1). The beta backend functions were pretested by providing 5 physicians with 4 different precise test tasks containing a total of 20 different subobjectives. Time limits and 40 acceptance criteria regarding “passing” or “nonpassing” of the targets were defined a priori. If the allotted time of 10 minutes per task expired before reaching the test target, the attempt was considered as *not passed*.

All included physicians worked with computers in their daily practice but did not have profound IT knowledge. A short feedback session was held after each task, allowing the physician to suggest improvements or to ask follow-up questions. If there were breaks or difficulties in the operating flow, the specific underlying reason was explored (Multimedia Appendix 2). All findings of the feedback rounds were systematically included in the audit protocol. Data were saved and then transferred into a Word (Microsoft Corp) document.

**Figure 3:**
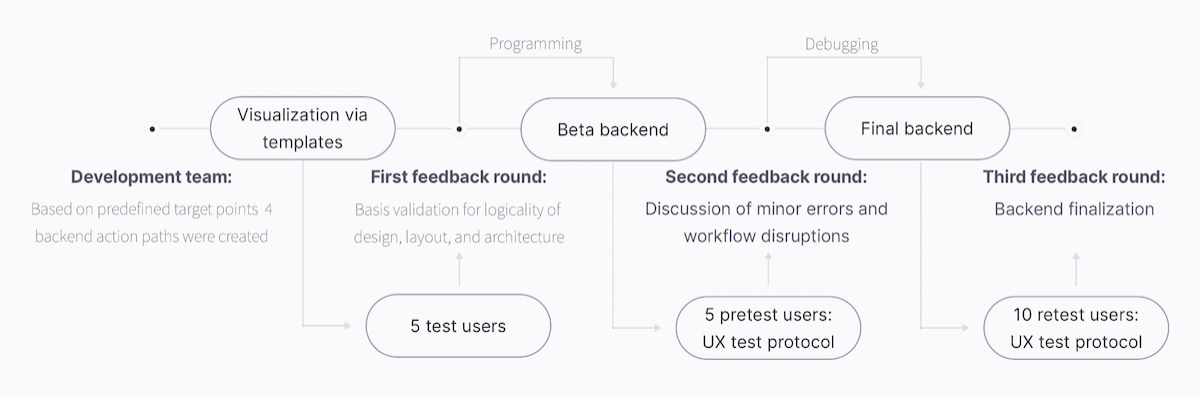
UX optimization and evaluation process. UX: user experience.

Again, all change requests and issues of the 5 pretest runs were discussed in the second feedback round within the development team, and improvement of the backend beta version was initiated. The required time spans for the given tasks in the final backend version were obtained again for 10 retest users to reevaluate the UX optimization success (Figure 3).

#### UX Optimization Tools

An in-screen frontend simulation provides the backend user with a preview of modifications made. This UX optimization tool provides a user-friendly visual interface and feedback mechanism between the back- and frontends (Figure 2).

An onboarding system was established to facilitate it for first-time users to get started using the backend. The user is guided step by step through the individual functions and necessary action paths by means of pop-up explanatory information.

### Ethical Considerations

All investigations with human participants were carried out with the consent of the Ethics Committee of the University of Duisburg-Essen (18-8142-BO), in accordance with national laws and in accordance with the tenets of the Declaration of Helsinki of 1975 (in the current, revised version).

## Results

### UX

In total, 1 female and 4 male physicians (mean age 39.2, SD 39.2, range 32-46 years) took part in the UX pretesting. All pretest participants were able to complete the tasks in the given time frame. On average, pretest users needed 3.33 (SD 1.05) minutes for the first, 4.04 (SD 0.40) minutes for the second, 7.14 (SD 1.34) minutes for the third, and 5.19 (SD 1.25) minutes for the fourth test flow for completion. The basic beta backend concept with the respective layout, menu navigation, wording, design, and color scheme was understandable to all participants. The handling was intuitive to learn with a few exceptions. If workflow interruptions occurred, the causes were clearly identified in the feedback rounds. There were some minor errors in the backend during the test rounds. Time saving was shown to be the top priority for backend usage (Multimedia Appendix 2).

**Figure 4:**
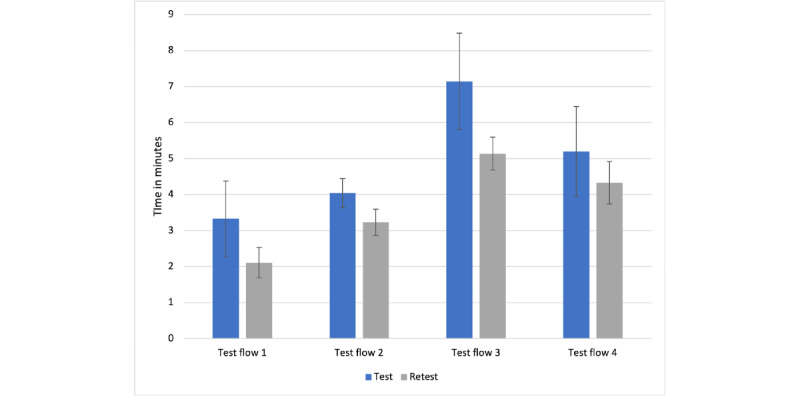
Test flow completion time. On average, 5.03 (summarized SD 1.07) minutes for the test run. In the retest, on average, 3.50 (summarized SD 0.45) minutes for the test run.

In the retest, 10 physicians needed on average 2.11 (SD 0.42) minutes for the first, 3.23 (SD 0.36) minutes for the second, 5.14 (SD 0.46) minutes for the third, and 4.33 (SD 0.59) minutes for the fourth test flow completion (Figure 4) and were aged 31-59 (mean 42, SD 9) years.

### DCMS-Based App Prototypes

#### Infectiology Guidelines

InfectioApp provides a compact guideline on the appropriate use of antibiotics and other anti-infective agents for the treatment of human infectious diseases. The guideline addresses physicians as well as other MPs and was created by the Saarland InfectioSaar Network in collaboration with the Antibiotic Stewardship Team at Saarland University Medical Center, Homburg, southwest Germany.

**Figure 5:**
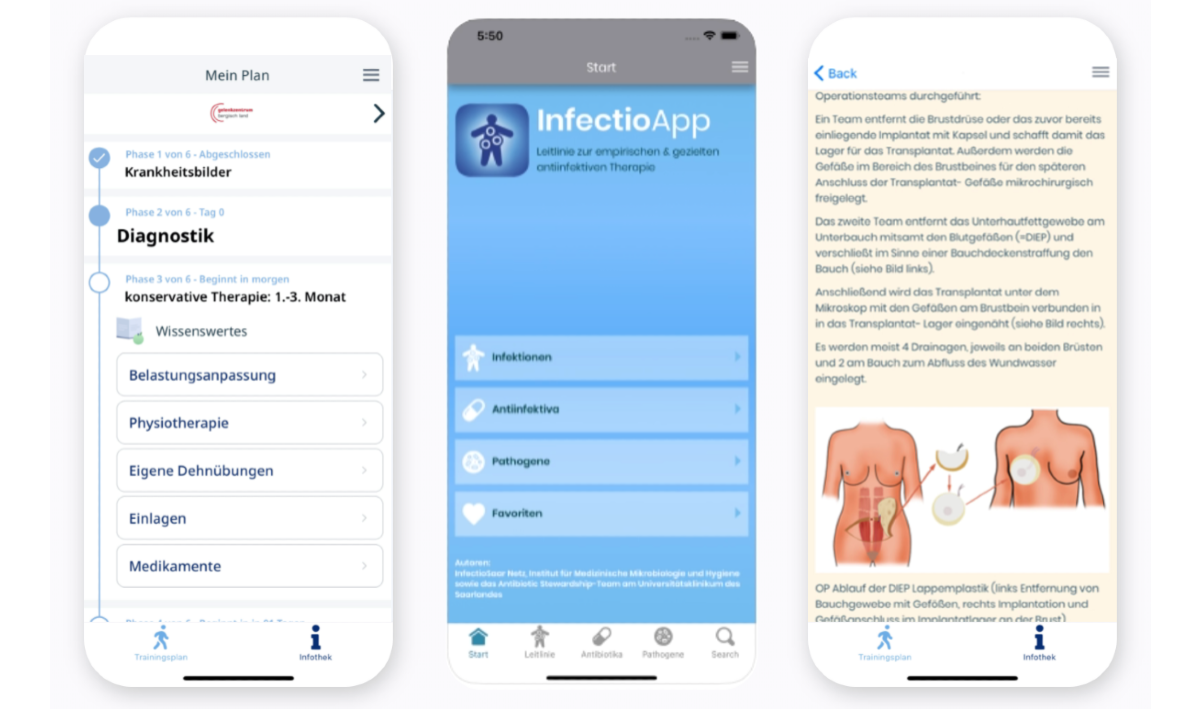
Data content management system–based apps in orthopedics, infectiology, and plastic and reconstructive surgery.

In addition to diagnostic and treatment recommendations, InfectioApp provides background information regarding important pathogens and clinical symptoms, and detailed guidance on appropriate dosing of anti-infective drugs in patients with renal or hepatic insufficiency. References to further literature are provided within the guideline. InfectioApp’s content corresponds to approximately 200 DIN A4 pages and is updated regularly (Figure 5).

#### Plastic and Reconstructive Surgery

The Fachklinik Hornheide App was specifically developed for a plastic, reconstructive, and aesthetic surgery department. Targeting patients, this app provides an overview of surgical procedures, indications, pre- and postoperative data, and general information about the hospital and the respective hospitalization.

To assess the specific needs of the patients, a survey about wishes and requirements was performed in the outpatient clinic before creation of the app (Figure 5).

#### Orthopedics

The backend was used to generate 3 different therapy plans for conservative or surgical therapy of forefoot and hindfoot disorders, based on the latest related medical literature and national guidelines. Special efforts have been made to ensure that communication of information is concise, clear, and easy to understand. With the cooperation of physiotherapists, a simple training program was created, which can be carried out without special equipment. A total of 15 exercises were made available to patients via the app. A special focus in this training circle was placed on early functional mobilization and stretching exercises (Figure 5).

## Discussion

### Principal Findings

Our study illustrates that backend integration offers great potential as an effective tool for app development in the mHealth sector. Specifically, backend operability for physicians and the implementation of end user–operable backends tend to be the key functions. The basic backend concept turned out to be clear to all test users, and its handling was intuitive to learn. Besides ease of usage, time saving was another key factor for backend usage. By establishing a practicable UX evaluation test protocol for the first time, we provide a basis for well-structured future software UX evaluation. Reasons for workflow interruptions in backend use have been identified and eliminated in the UX evaluation test. Finally, the developed backend was tested for its success on the basis of several pilot apps.

### Backend Infrastructures in mHealth

For now, the rollout of digital health apps has not led to the intended disruption of widespread smartphone implementation in the German health care sector. The reasons for limited availability of apps on prescription could not yet be determined, but opinions have been expressed that neglecting MPs in the app development process is one of the main reasons [15]. If MPs had access to the content and functions of apps, mHealth apps could be adapted to the individual needs of patients and MPs within predefined limits.

The benefits of backend usage are not new and represent an established method for processing large data sets in a software architecture. In the medical field, there have been some innovative approaches that have taken advantage of the high scalability and flexibility of backends [21-24]. An adaptive approach was proven to be promising for therapy support of chronically ill patients. This developed system consists of cross-platform client and caregiver apps, a web-based clinician portal, and a secure communication protocol, all supported by a backend server [20]. However, the evidence and knowledge base of the technical development process and clinical implementation is rather weak.

It is debatable whether using “off-the-shelf” apps is at all feasible. In this context, the need remains for customized software that addresses individual use cases rather than one-size-fits-all solutions [25]. However, there are some risks and disadvantages that need to be considered when using end user–operable backends. The time factor, as mentioned in the UX evaluation test, seems the most challenging factor in motivating MPs to use an innovative software solution. From this perspective, building up databases with a wide variety of media and content might facilitate frontend designing, since the user only has to select and not create new content. Collaboration with medical societies could generate guideline-compliant, reputable content and make it publicly available in the DCMS as a shared value approach [26,27]. We hereby clearly demonstrate that time-consuming creation of guideline content, in line with current valid evidence, takes place only once. Additional hospitals considering to use the app could add further references and minor adaptations; for example, local antibiotic resistance.

Backend-based stand-alone apps with individual functions, a unique app logo, and the implementation of individual corporate designs might be developed more efficiently than the alternative of “starting from scratch” with every app. An even simpler alternative could be a contentless home app with predefined functions that communicate with the backend. This content could be adapted to the respective requirements via the backend and be transferred to the home app via QR codes or links, which could then personalize the app. Ensuring a high quality of the generated content remains challenging, and the establishment of a quality management system has to be mandatory.

This highly effective concept also allows focusing on small, financially less lucrative pathologies such as a hallux valgus—contrary to greater financial interests by established companies.

### Usability

Usability is a quality attribute that measures the user’s interface handling. In software, usability is a necessary basic condition to survive [28]. Based on a recent study among German physicians, intuitive usability was considered the most important factor for software quality and acceptance [18]. Evidence for usability compared to the exponentially rising rating for medical apps in the app stores for quality, and especially usability evaluation, is scarce but growing [29-32]. The implementation of complex functions in user-friendly interfaces poses challenges and directly contrasts our data and other researchers’ results, demonstrating that usability can be optimized effectively with simple measurements [33]. Our data show a decreased workflow completion time from 5.03 minutes to 3.50 minutes. Additionally, the decreased summarized SD (test SD 1.07 vs retest SD 0.45) might be interpreted as an indicator for decreased usage hurdles in the backend compared to those before usability optimization. It can be concluded that usability is experienced more homogeneously even in a larger group. Testing for significance was not carried out due to the small test sample, but the tendency is evident.

In this context, the development and reevaluation process in a multidisciplinary team was proved to be crucial. It was value-adding to see how the different perspectives and foci of medical professionals, developers, and usability specialists on software usability diverged and finally merged into one vision. In conclusion, the importance of the discourse among individual perspectives must be emphasized.

### Limitations

This study has some limitations. The backend’s usability optimization process was evaluated as a practical approach. However, it is based on principles that have already been established decades ago in other fields. Nevertheless, these aspects are relatively new in the health sector [34]. Validation of the usability optimization test using an already established usability test—for example, the System Usability Scale—is still in progress [35]. However, it has already been shown that in clinical use of the system, apps could be effectively developed with the involvement of physicians. Moreover, the backend’s usability was only evaluated by a relatively small number of test users. However, the study protocol is based on the Nielsen postulate, which states that 85% of usability errors can be identified within 5 test users [36]. Elimination of the remaining 15% of errors would mean a significant additional effort, though an increased sample size is obviously associated with improved usability testing [37]. Economic aspects of the development process were not considered in detail. A comparison of backend-based development costs with conventional app developments is needed.

### Study Strengths

The advantages of the study are that a practicable procedure, based on existing theoretical approaches, of backend and UX optimization is documented and made available for other app developers and developing teams. Furthermore, the principle and functionality of backend integration in medical apps is described. Evidence regarding the usability and benefits of backends in combination with health apps is rudimentary. Thus, the study provides valuable insights into the participation of medical staff and the value of backends in the development of health apps.

### Outlook

Important cornerstones of digital technologies are flexibility and adaptability. An increase in the knowledge and progress in the field of medicine is rapid, so mHealth apps must also be able to transfer this pace into everyday clinical practice. With the introduction of every new innovative technology, usability plays a decisive role in whether the technology is ultimately used and can establish itself. Backend structures could be the next compelling step in the evolution of promising mHealth solutions, both technically and economically, as these enable the practical involvement of health care professionals in the app functionality and content configuration. However, comparative studies are required to gain funded evidence regarding the systems’ effects on treatment progress in comparison with established nondigital therapy paths to reach a final scientific conclusion.

### Conclusions

Backend operability for physicians offers great potential as an effective tool for the development of apps in the mHealth sector. A sophisticated and time-saving UX emerged as the top priority for medical software usage. Basic interventions are sufficient for adequate UX optimization. UX evaluation of practicable, well-structured software is possible based on the UX evaluation test protocol.

## Appendix

Multimedia Appendix 1 User Experience Evaluation Test.

Multimedia Appendix 2 User Experience Evaluation Test (backend beta version).

## Abbreviations

DCMS: data content management system
mHealth: mobile health
MP: medical professional
UX: user experience

## Data Availability

The data sets generated and analyzed during the current study are included in this published article and its supplementary information files or available from the corresponding author on reasonable request.
