# Supplementary material for: Development of Open Backend Structures for Health Care Professionals to Improve Participation in App Developments: Pilot Usability Study of a Medical App
Source: JMIR Form Res. 2023 Apr 13;7:e42224. doi: 10.2196/42224 (PMC10141301; doi:10.2196/42224)
Supplement: Multimedia Appendix 1 [file formative_v7i1e42224_app1.docx]

User Experience Evaluation Test

# **1. Pre-testing**

## **1.1 Test description**

Test object: *Object’s name*

### Testflow 1: *Testflow’s name*

### *Describe/define all tasks/functions as precise as possible.*

### Testflow 2: …

### Test users are asked to (a) …, (b) …, and (c) …

## Number of test users: *(5 is the most efficient amount of test users)*

## Duration: *per test*

## Period: …

## Tester: …

## **1.2 User Experience Evaluation Test objective**

### *Define and name the test objectives as precise as possible.*

## **1.3 Acceptance criteria**

### *Define and name the test acceptance criteria as precise as possible.*

**Testflow 1:** *Testflow’s name*

### Passed: *Describe/define all passing-criteria as precise as possible.*

Not passed: *Describe/define all non-passing-criteria as precise as possible.*

**Testflow 2:** …

Passed: Test users can (a) …, (b) …, and (c) …

Not passed: Test users cannot (a) …, (b) …, and (c) …

# **2. Test execution**

## **2.1 Test setting**

*A possible test scenario: Via video conference call software using screen sharing (test-user's point of view) and screen capture recording. The tester asks the test user to start the relevant software and instructs the user to carry out the mentioned tasks (1.1). Test user is encouraged to think aloud and voice their every opinion.*

## **2.2 Test results**

**Test user 1:** *Test user’s name and further information.*

Date of the test: …

Test flow 1 (passed/not passed): *Define if the test-user fulfills the a priori defined passing/non-passing-criteria.*

What I would like to improve: *Use feedback rounds to document improvement suggestions and identify underlying causes if passing-criteria were not met.*

Test flow 2 (passed/not passed): Test user 1 was able/not able to...

What I would like to improve: …

## **2.3 Test result interpretation**

*Use the video recordings to assess the acceptance criteria for passing or non-passing. Include all findings of the feedback rounds systematically in the audit protocol*

# **3. Measures**

*Discuss the audit protocol with all members of the development team to obtain a multidisciplinary response. Afterwards, the software implementation can be started.*
