# Supplementary material for: Development of Open Backend Structures for Health Care Professionals to Improve Participation in App Developments: Pilot Usability Study of a Medical App
Source: JMIR Form Res. 2023 Apr 13;7:e42224. doi: 10.2196/42224 (PMC10141301; doi:10.2196/42224)
Supplement: Multimedia Appendix 2 [file formative_v7i1e42224_app2.docx]

User Experience Evaluation Test

# **1. Backend Pre-testing (wording, usability)**

## **1.1 Test description**

Test object: backend beta version (https://backend.mediploy.com/login)

### Testflow 1: Creation of an application

### Test users are asked to (a) create, (b) duplicate, and (c) delete a new plan (application). Test users are asked to retrieve the corresponding QR code and plan print version.

### Testflow 2: Plan Onboarding

### Test users are asked to create the corresponding plan's onboarding and provide their own "onboarding" definition.

### Testflow 3: Plan development

### Test users are asked to create 2 plan phases (Phase 1: Operation Day, Day 0; Phase 2: Week 1-2, Day 1-14) and add modules to each phase. Test users are asked to (a) edit, (b) duplicate, (c) move, and (d) delete modules inserted in the plan.

### Testflow 4: Module template creation

### Test users are asked to create a module template with a corresponding icon and text, add a table and an image, and give their own "module template" definition. Test users are asked to add a table and an image to the module template.

## Number of test users: 5

## Duration: 30-60 minutes each

## Period: July and August 2020

## Tester: FD

## **1.2 Test objective**

The aim of the pre-test is to obtain information on the usability of the backend adjustments in the beta version.

## **1.3 Acceptance criteria**

The following acceptance criteria are defined for evaluating the new content and functions for the individual testflows:

**Testflow 1: Creation of an application**

Passed: Test users can quickly (a) create, (b) duplicate, and (c) delete a new application. Test users can retrieve the corresponding QR code and plan print version.

Not passed: Test users cannot (a) create, (b) duplicate, and (c) delete a new application. Test users cannot retrieve the corresponding QR code and plan print version. The task is also not passed if it takes more than 10 minutes to complete.

**Testflow 2: Plan Onboarding**

Passed: Test users can access the onboarding menu of the corresponding plan without delay and create or change the onboarding content and structure. Test users can provide their own adequate definition of the term "onboarding".

Not passed: Test users cannot quickly access the onboarding menu of the corresponding plan and create or change the content and structure of the onboarding. Test users cannot provide their own adequate definition of the term "onboarding". The task is also not passed if it takes more than 10 minutes to complete.

**Test flow 3: Plan creation**

Passed: Test users can create 2 plan phases (Phase 1: Operation Day, Day 0; Phase 2: Week 1-2, Day 1-14) and fill each with modules. Test users can (a) edit, (b) duplicate, (c) move, and (d) delete modules inserted in the plan.

Not passed: Test users cannot create 2 plan phases (Phase 1: Operation Day, Day 0; Phase 2: Week 1-2, Day 1-14) or fill each of them with modules. Test users cannot (a) edit, (b) duplicate, (c) move, and (d) delete modules inserted in the plan. The task is also not passed if it takes more than 10 minutes to complete.

**Testflow 4: Module Template Creation**

Passed: Test users can create a module template with a corresponding icon and text, add a table and an image, and provide their own definition of the term "module template". Test users can add one table and one image to each module template.

Not passed: Test users cannot create a module template with a corresponding icon and text, add a table and an image, or provide their own definition of the term "module template". Test users cannot add a table and an image to the module template. The task is also not passed if it takes more than 10 minutes to complete.

# **2. Test execution**

## **2.1 Test results**

**Test user 1: Trauma surgeon, 34 years**

Date of the test: 22.07.2020

Test flow 1 (passed): Test user 1 was able to complete the given tasks quickly and without any problems.

What I would like to improve: -

Testflow 2 (passed): Test user 1 was able to complete the given tasks quickly and without any problems. However, the term "onboarding" could not be precisely defined.

What I would like to improve: Use different term for the word "onboarding". It is not clear.

Testflow 3 (passed): Test user 1 was able to complete the given tasks quickly and without problems. It took a lot of time to find other module template categories.

What I would like to improve: Clicking on the module (icon) in the plan, the content should be automatically displayed as a preview. The fact that one must select the edit button is a break of logic and inefficient.

Testflow 4 (passed): Test user 1 was able to complete the given tasks quickly and without problems.

What I would like to improve: Accessing a separate chapter for this is cumbersome.

**Test user 2: Senior trauma surgeon, 46 years**

Date of test: 01.08.2020

Test flow 1 (passed): Test user 2 was able to complete the given tasks quickly and without problems.

What I would like to improve: -

Testflow 2 (passed): Test user 2 was able to complete the given tasks quickly and without any problems. The term "onboarding" could be defined, but the context was not fully clear.

What I would like to improve: Use a different term for the word "onboarding". It is not understandable.

Testflow 3 (passed): Test user 2 was able to complete the given tasks quickly and without problems. The operation was intuitive to learn, no explanation by the test leader was necessary.

What I would like to improve: All templates and plans must be already available in the system. It should not take much time to use the system. "If I am already stressed all day, I don't want to waste additional time in front of the PC".

Testflow 4 (passed): Test user 2 was able to complete the given tasks quickly and without any problems.

What I would like to improve: It is inefficient that one must change the tab just to be able to create module templates. "If the templates are not already there, I would never use the system. I would only make small changes to the templates at most."

**Test user 3: Senior orthopedic, 41 years**

Date of test: 02.08.2020

Testflow 1 (passed): Test user 3 was able to complete the given tasks quickly and without any problems.

What I would like to improve: The print version's graphic design needs some improvement (layout), the clinic icon does not properly fit into the corresponding field (too small).

Testflow 2 (passed): Test user 3 was able to complete the given tasks quickly and without problems. The term "onboarding" could be defined, but the context was not clear.

What I would like to improve: Use a different term for the word "onboarding". It is not understandable. Especially it is not clear that the patient (end user of the app) is onboarded. In general, all explanatory texts in the backend should be worded more clearly according to grammar and the texts themselves. The onboarding of the backend itself is great, but the texts should also be revised.

Testflow 3 (passed): Test user 3 was able to complete the given tasks quickly and without problems. The operation was intuitively learnable, few explanations had to be given by the test leader. The logic between modules and phases took some time. The selection option of the module categories was only found after prompting.

What I would like to improve: Time saving! It would be good if it would be clearer that modules can be assigned to phases (e.g. phase is at the top, modules below. Or phase on the left and modules on the right). Perhaps the buttons "Phase" and "Modules" could also be graphically adapted to the representation in the plan. Module category selection must stand out more.

Testflow 4 (passed): The test user 3 was able to complete the given tasks quickly and without problems.

What I would like to improve: It is inconvenient that one must change the tab just to be able to create module templates.It must be possible to load modules into the system as module templates from the plan generation.

**Test user 4: Plastic and reconstructive surgeon, 32 years**

Date of test: 06.08.2020

Test flow 1 (passed): Test user 4 was able to complete the given tasks quickly and without any problems.

What I would like to improve: Possibly change the word "application".

Testflow 2 (passed): Test user 4 was able to complete the given tasks quickly and without problems. The term "onboarding" could be defined, but the context was not clear.

What I would like to improve: Use a different term for the word "onboarding", e.g. "tutorial". It is better understandable.

Testflow 3 (passed): Test user 4 was able to complete the given tasks quickly and without problems. The operation was intuitively learnable, no explanation by the test leader was necessary. However, further categories of the module templates were only found after prompting. The "badge" function was not clear and had to be explained. The plan naming function was too small and could only be found after prompting.

What I would like to improve: The drop-down arrow for module category templates should be larger. Badge function needed? If so, choose other term and explain! Plan naming function needs to be more dominant.

Testflow 4 (passed): Test user 4 was able to complete the given tasks quickly and without problems.

What I would like to improve: It is inconvenient that you must change the tab just to be able to create module templates.

**Test user 5: Head orthopedics/trauma surgeon, 43 years old**

Date of testing: 06.08.2020

Testflow 1 (passed): Test user 5 was able to complete the given tasks quickly and without any problems. The QR code creation function was not immediately recognizable by the icon.

What I would like to improve: -

Testflow 2 (passed): Test user 5 was able to complete the given tasks quickly and without problems. The term "onboarding" could be defined, but the context was not clear.

What I would like to improve: Specific things could be written into the empty text fields that should be implied in the onboarding screen.

Testflow 3 (passed): Test user 5 was able to complete the given tasks quickly and without problems. The handling was intuitive to learn, there was no need for much explanation by the test leader. It took a little time to add modules to the plan. The plan naming was not easy to find.

What I would like to improve: If a new application was created, it is mandatory to name it as the first step. Only then it should be possible to edit the plan. In the whole process of creation, saving time is the top priority!

Testflow 4 (passed): Test user 5 was able to complete the given tasks quickly and without problems.

What I would like to improve: It is inconvenient that one must change the tab just to be able to create module templates. At the end of a completed plan generation, one could query whether the plan should be uploaded to the system, which would create a database.

## **2.2 Test result interpretation**

1. The basic principle was clear to all test participants.
2. With a few exceptions, the backend’s operation was intuitive to learn.
   1. Wording:
      1. onboarding (mentioned 5x), application (1x).
      2. Explanation texts in the backend onboarding must be formulated more clearly, grammar and spelling must be corrected
   2. Usability:
      1. Preview view of the content of each module in the plan
      2. Selection module template category needs to be highlighted more clearly
      3. Module templates must become easier to create
      4. Plan print version should be revised (clinic icon too small, improved text formatting)
      5. Reorganization of the buttons "add module" and "add phase
      6. Definition of the date-defining phase (day 0) as first step, remaining phases are related to it
      7. Re-evaluation of the badge function (delete vs. explain)
      8. Re-evaluation of the plan-naming-function (first mandatory step in plan generation)
      9. Onboarding masks could contain concrete prompts in addition to sample texts
3. There occurred some small bugs in the backend during the test phase.
4. Top priority in the use of the backend needs to be time saving.
   1. Establish a plan-upload-function for all users of the backend (plan database)

# **3. Measures**

All mentioned aspects (2.2) have been incorporated in the final backend redesign.

|  | **Testflow 1** | **Testflow 2** | **Testflow 3** | **Testflow 4** |
| --- | --- | --- | --- | --- |
| **Test user 1** | 2.16 | 3.42 | 6.43 | 4.23 |
| **Test user 2** | 2.32 | 3.11 | 6.17 | 4.44 |
| **Test user 3** | 3.55 | 4.05 | 7.23 | 5.54 |
| **Test user 4** | 3.46 | 3.59 | 7.33 | 5.46 |
| **Test user 5** | 3.14 | 4.03 | 8.56 | 6.30 |

Table 1 Required time in minutes and seconds to accomplish the respective testflow (primary test).

|  | **Testflow 1** | **Testflow 2** | **Testflow 3** | **Testflow 4** |
| --- | --- | --- | --- | --- |
| **Test user 6** | 2 | 3.06 | 5.23 | 3.59 |
| **Test user 7** | 2.11 | 3.11 | 5.03 | 4.11 |
| **Test user 8** | 1.58 | 2.55 | 5.12 | 4 |
| **Test user 9** | 2.06 | 3.11 | 5.34 | 4.59 |
| **Test user 10** | 2.18 | 2.51 | 5.04 | 5.13 |
| **Test user 11** | 2.10 | 2.46 | 5.18 | 3.56 |
| **Test user 12** | 2.24 | 3.01 | 4.59 | 4.03 |
| **Test user 13** | 1.58 | 2.45 | 5.09 | 4.54 |
| **Test user 14** | 3.11 | 3.48 | 6.22 | 5.32 |
| **Test user 15** | 2.14 | 2.58 | 4.55 | 4.41 |

Table 2 Required time in minutes and seconds to accomplish the respective testflow (retest).
